# The association between polymorphisms in the *PDCD1* gene and the risk of cancer: A PRISMA-compliant meta-analysis

**DOI:** 10.1097/MD.0000000000004423

**Published:** 2016-10-07

**Authors:** Jie Zhang, Taiqiang Zhao, Chengjie Xu, Jiang Huang, Hua Yu

**Affiliations:** a Department of Laboratory Medicine; b Department of Respiratory Medicine, Sichuan Academy of Medical Sciences and Sichuan Provincial People's Hospital, Chengdu, Sichuan, China.

**Keywords:** cancer, meta-analysis, PDCD1, polymorphism

## Abstract

**Background:**

The effects of the programed cell death 1 (*PDCD1*) gene polymorphisms on cancer risk have been investigated in some studies; however, the results were conflicting and ambiguous. Therefore, we aimed to do a meta-analysis to investigate the association of PDCD1 polymorphisms with cancer risk from all eligible case–control studies.

**Materials and methods:**

An electronic search of the PubMed, Embase, Chinese National Knowledge Infrastructure, and Wanfang databases was performed. The association between *PDCD1* polymorphisms with cancer risk was calculated with odds ratios (ORs) and their corresponding 95% of confidence intervals (CIs).

**Results:**

A total of 24 case–control studies from 13 articles that investigated the associations of 5 widely studied polymorphisms in *PDCD1* gene and cancer risks were included. The results of meta-analysis: the PDCD-1.5 (rs2227981) and PDCD-1.3 (rs11568821) polymorphisms were associated with decreased risk of cancer (rs2227981: OR = 0.75, 95% CI: 0.64–0.86, *P* < 0.0001 for TT vs TC + CC; rs11568821: OR = 0.79, 95% CI: 0.65–0.96, *P* = 0.02 for TC vs TT), while no significant associations were found for the other 3 polymorphisms (PDCD-1.9 [rs2227982] polymorphism: OR = 1.03, 95% CI: 0.90–1.18, *P* = 0.66 for CC + TC vs TT; *PDCD1* rs7421861 polymorphism: OR = 1.10, 95% CI: 0.96–1.25, *P* = 0.16 for CC + TC vs TT; PDCD-1.6 [rs10204525] polymorphism: OR = 0.93, 95% CI: 0.82–1.05, *P* = 0.24 for GG + GA vs AA).

**Conclusion:**

The meta-analysis suggests that the PDCD-1.5 (rs2227981) and PDCD-1.3 (rs11568821) polymorphisms are associated with susceptibility of cancer. Further studies with larger sample sizes are required to make a better assessment of the above association.

## Abstract 2

Supplemental Digital Content is available in the text

## 1 Introduction

Programed cell death-1 (*PDCD1*) is an immunoreceptor belonging to the CD28/CTLA-4 family.^[1]^ It is a 55-kd types I transmembrane glycoprotein and a member of the immunoglobulin superfamily B7.^[2–4]^ It is expressed on activated B cells, T cells, and monocytes, and its ligand (PD-L) on immune and nonimmune cells including tumor cells.^[5]^ PD-1 was first identified by Ishida in 1992,^[6]^ its function of negatively regulation in immune response was later found by the generation of *PDCD1*^*−/−*^ mice.^[7]^ PD-1 is involved in almost every aspect of immune responses including autoimmunity, tumor immunity, infectious immunity, transplantation immunity, allergy, and immunological privilege.^[1]^ The human *PDCD1* gene is located on 2q37.3. In the *PDCD1* gene, several polymorphisms have been identified, such as PDCD-1.1 (rs36084323), PDCD-1.3 (rs11568821), PDCD-1.5 (rs2227981), PDCD-1.9 (rs2227982), and so on.^[8–10]^ The association between polymorphisms in *PDCD1* gene and cancer risk has been studied in many studies. However, these associations were still inconclusive.^[8–13]^ Although a meta-analysis reported the association between PDCD-1.5 (rs2227981) polymorphism and the risk of cancer^[14]^; however, they only reported 1 polymorphism and did not report the exact search date. The association between other polymorphisms with cancer risk should also be assessed. Thus, we conducted a comprehensive meta-analysis to investigate the association of *PDCD1* gene polymorphisms and cancer risk.

## 2 Materials and methods

The Preferred Reporting Items for Systematic Reviews and Meta-Analyses statement was used in the process of the meta-analysis (table S1).^[15]^ The present study is a meta-analysis, and ethical approval was not necessary.

### 2.1 Literature search

A literature search of the PubMed, EMbase, Chinese National Knowledge Infrastructure, and WanFang databases was carried out to collect the case–control studies that investigated the association between polymorphisms of *PDCD1* gene and the risk of cancer. The date was extended to December 10, 2015. The search words were as follows: polymorphism, variant, cancer, carcinoma, PDCD1, and programed death-1.

### 2.2 Inclusion and exclusion criteria

We selected eligible studies according to the following criteria: case–control studies, investigating the association between the PDCD1 polymorphisms and cancer risk, detailed genotype data for estimating of odds ratio (OR) and 95% confidence interval (CI), and articles written in English or Chinese. Exclusion criteria were the following: insufficient information on the distribution of *PDCD1* genotypes, case-only studies, and duplicated publications. If multiple studies had overlapping or duplicate data, only those with complete data were included.

### 2.3 Data extraction

Data extraction was performed independently by 2 of the authors (JZ and TZ) using a standard protocol according to the inclusion criteria. The following data were extracted: the name of the first author, year of publication, country of participants, ethnicity, genotyping methods, and genotype distribution of cases and controls. Disputes were settled by discussion.

### 2.4 Statistical analysis

Any polymorphism studied in at least 3 case–control studies was included for data analysis. Crude ORs with 95% CIs were calculated to evaluate the strength of the association between *PDCD1* polymorphisms and cancer risk.^[16,17]^ All genetic models (additive, dominant, recessive, and codominant) were used to assess the association.^[17,18]^ Take the PDCD-1.9 (rs2227982) polymorphism as an example, the genetic models were as follows: additive model (C vs T), dominant model (CC + CT vs TT), recessive model (CC vs TT + CT), and codominant model (CC vs TT, CT vs TT). A statistical test for heterogeneity was performed based on the *Q* statistic.^[19]^ If *P* < 0.10 for *Q* test suggested significant heterogeneity, then the random effects model was conducted to calculate the pooled OR; otherwise, the fixed effects model was selected.^[18,20]^ Sensitivity analysis was performed by omitting each study in turn to assess the quality and consistency of the results. Begg funnel plot and the Egger test were used to evaluate possible publication bias of literatures.^[21]^ All statistical tests were performed by using Revman 5.3 software (The Cochrane Collaboration, UK) and STATA 12.0 software (Stata Corporation, College Station, TX). *P* values <0.05 were considered statistically significant.

## 3 Results

### 3.1 Eligible studies

**Figure 1:**
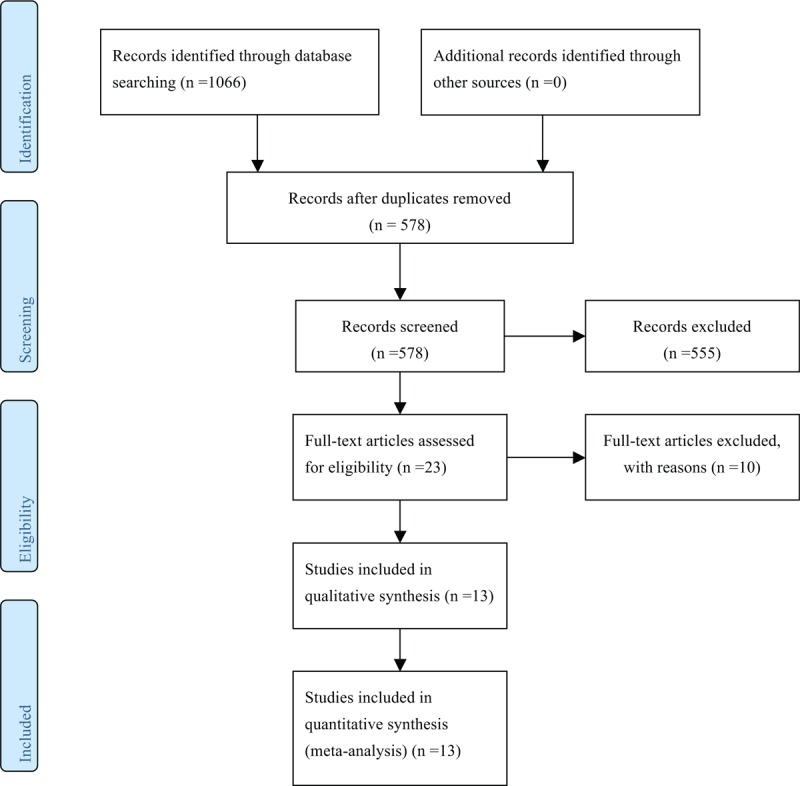
Selection of studies for inclusion in meta-analysis.

We initially identified 1066 potentially relevant studies after searching the databases. After excluding the duplicated records, 578 studies were left for screening. After reading the title and the abstracts of these studies, 555 studies were excluded for not reporting the association between the *PDCD-1* polymorphisms and cancer risks reviews. Thus, 23 studies were left for full-text assessment and data extraction. Among these studies, 2 studies were excluded for not reporting useful data for meta-analysis, 3 were excluded for not being case–control studies, and 5 were excluded for not reporting polymorphism in more than 3 case–control studies. Thus, 13 studies that met the predescribed inclusion criteria were included in the meta-analysis of the association between *PDCD1* polymorphisms and cancer risk (Fig. 1).^[2–5,8–13,22–24]^ Characteristics of all eligible case–control studies are summarized in Table 1. There were 7 case–control studies on PDCD-1.5 (rs2227981) polymorphism,^[2,3,5,8,10,12,24]^ 5 on PDCD-1.9 (rs2227982) polymorphism,^[2,5,9,11,23]^ 4 on rs7421861 polymorphism,^[2,9,11,23]^ 4 on PDCD-1.3 (rs11568821) polymorphism,^[5,13,22,24]^ and 4 on PDCD-1.6 (rs10204525) polymorphism,^[4,9,11,23]^ respectively. Of the 13 included studies, 7 types of cancers including gastric, breast, esophageal, liver (hepatocellular carcinoma), colorectal, cervical, and lung cancer were involved. The genotype distributions in the studies considered in the present meta-analysis are shown in Table 2.

**Table 1:**
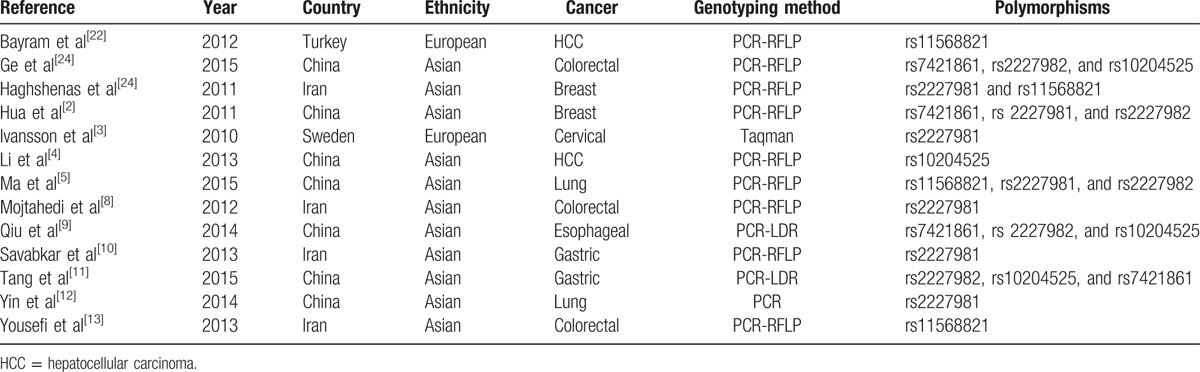
The characteristics of the included studies.

**Table 2:**
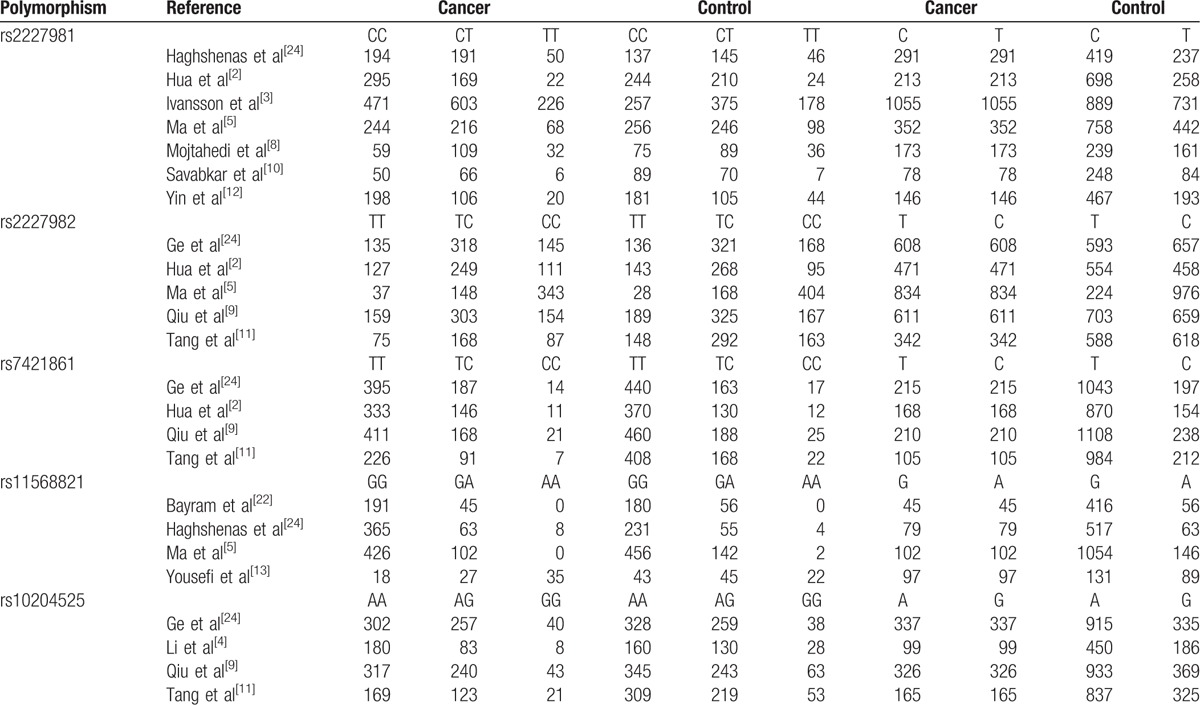
The genotypes and alleles distributions of included polymorphisms.

### 3.2 Meta-analysis results

**Figure 2:**
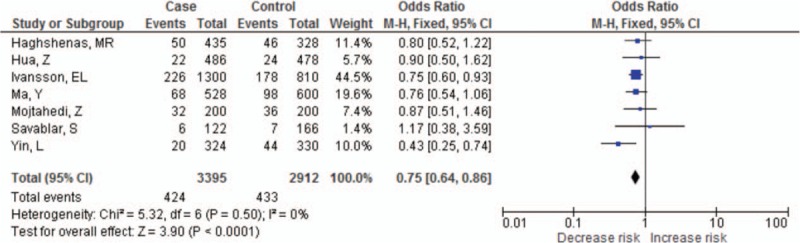
Meta-analysis of programed cell death-1.5 (rs2227981) polymorphism and cancer risk.

**Figure 3:**
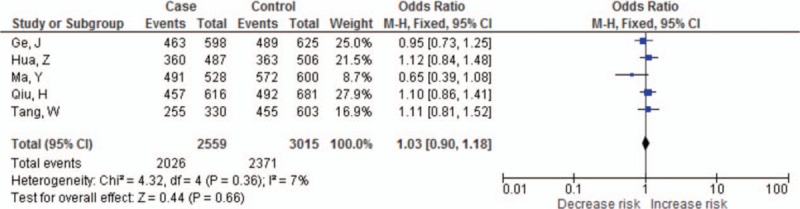
Meta-analysis of programed cell death-1.9 (rs2227982) polymorphism and cancer risk.

**Figure 4:**
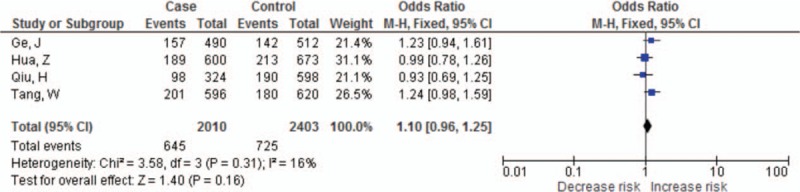
Meta-analysis of *PDCD1* gene rs7421861 polymorphism and cancer risk.

**Figure 5:**
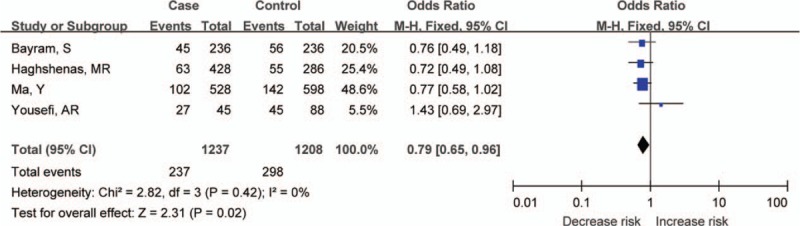
Meta-analysis of programed cell death-1.3 (rs11568821) polymorphism and cancer risk.

**Figure 6:**
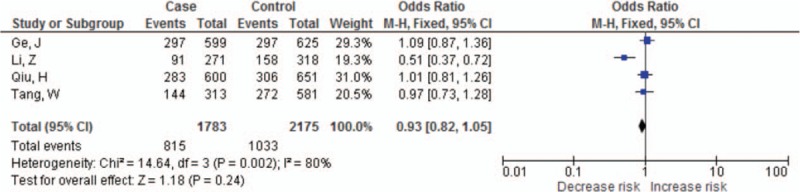
Meta-analysis of programed cell death-1.6 (rs10204525) polymorphism and cancer risk.

The summary results for the association between *PDCD-1* polymorphisms and the risk of cancer are shown in Table 3. For the PDCD-1.5 (rs2227981) polymorphism, we found a significant association between the polymorphism and overall cancer risk in the recessive genetic model (OR = 0.75, 95% CI: 0.64–0.86, *P* < 0.0001) (Fig. 2). For the PDCD-1.9 (rs2227982) polymorphism, there was no statistical evidence of an association between the polymorphism and overall cancer risk in the dominant genetic model (OR = 1.03, 95% CI: 0.90–1.18, *P* = 0.66) (Fig. 3). For the rs7421861 polymorphism, there was no statistical evidence of an association between the polymorphism and overall cancer risk in the dominant genetic model (OR = 1.10, 95% CI: 0.96–1.25, *P* = 0.16) (Fig. 4). For the PDCD-1.3 (rs11568821) polymorphism, there was statistical evidence of an association between the polymorphism and overall cancer risk in TC versus TT genetic model (OR = 0.79, 95% CI: 0.65–0.96, *P* = 0.02) (Fig. 5). For the PDCD-1.6 (rs10204525) polymorphism, there was no statistical evidence of an association between the polymorphism and overall cancer risk (OR = 0.93, 95% CI: 0.82–1.05, *P* = 0.24) (Fig. 6).

**Table 3:**
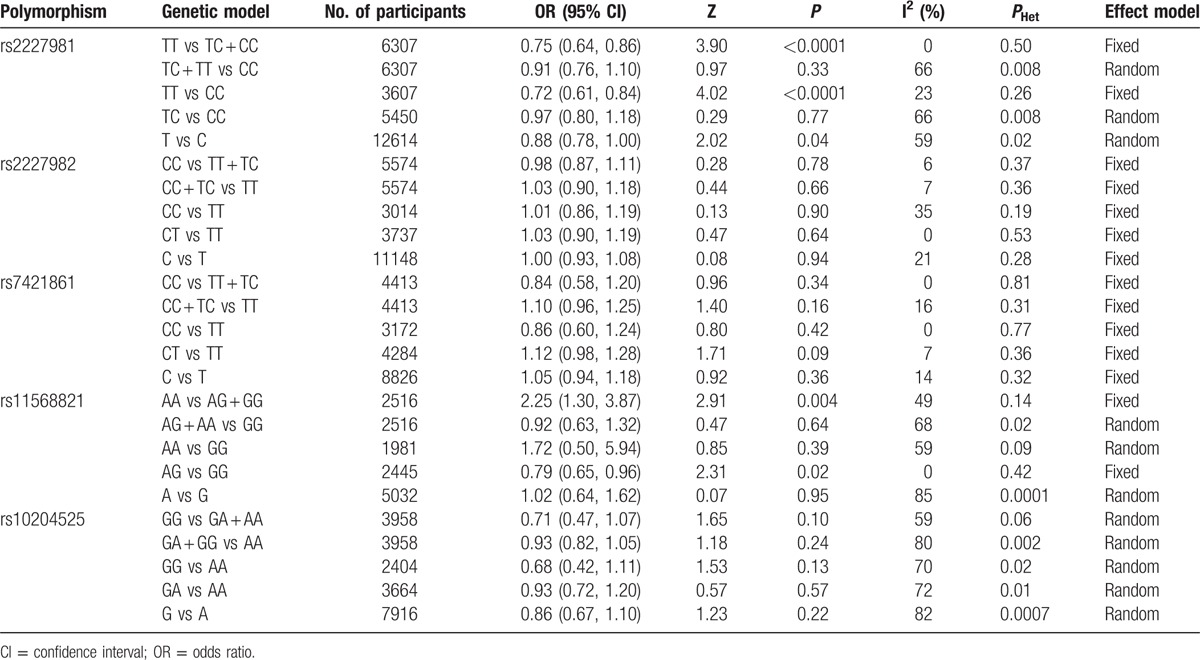
Summary of results from different comparative genetic models for each polymorphism.

### 3.3 Publication bias

Publication bias was analyzed by Begg and Egger tests for each polymorphism. No publication bias was detected with either the Begg funnel plot or the Egger test (PDCD-1.5 [rs2227981] polymorphism: Supplement figure 1, t = 0.26 and *P* = 0.804 for Egger test; PDCD-1.9 [rs2227982] polymorphism: Supplement figure 2, t = −2.37 and *P* = 0.098 for Egger test; rs7421861 polymorphism: Supplement figure 3, t = −0.37 and *P* = 0.744 for Egger test; PDCD-1.3 [rs11568821] polymorphism: Supplement figure 4, t = 1.77 and *P* = 0.220 for Egger test; PDCD-1.6 [rs10204525] polymorphism: Supplement figure 5, t = −2.98 and *P* = 0.097 for Egger test).

## 4 Discussion

Accumulative evidence suggests that PDCD1 is a negative regulator of the immune response.^[5,11,23]^ Genetic variants in *PDCD1* gene have been associated with the pathogenesis of cancers. Several important variants in the gene have been identified, such as the PDCD-1.5 (rs2227981) polymorphism, PDCD-1.9 (rs2227982), and so on.^[5,9]^ Up to now, the associations between polymorphisms in the *PDCD1* gene and the risk of cancer were still inconclusive; thus, we performed the current meta-analysis. To the best of our knowledge, this is the first comprehensive meta-analysis to assess the association of *PDCD1* gene polymorphisms with the risk of cancer.

The current meta-analysis, which included a total of 24 case–control studies from 13 articles, investigated the associations of 5 widely studied polymorphisms in *PDCD1* gene and cancer risk. The results indicated that the variant TT genotype of the rs2227981 polymorphism and TC genotype of the rs11568821 polymorphism were associated with significant decreased risk of cancer, whereas the other 3 polymorphisms (rs2227982, rs7421861, and rs10204525) did not appear to have a significant association with cancer risk. Previous studies reported that the PDCD-1.5 (rs2227981) polymorphism was an asynonymous mutation (C to T, Ala to Ala)^[2,24]^; it may influence the expression and function of *PDCD1* through linkage disequilibrium with other nucleotide polymorphisms in *PDCD1* gene or other nearby genes.^[2,24]^ Accordingly, the polymorphism may influence the susceptibility to cancer through these mechanisms.

In this meta-analysis, we also found that the PDCD-1.3 (rs11568821) polymorphism was significantly associated with decreased risk of cancer, and the genotype TC might be a risk factor. A possible reason might be that this polymorphism (T to C) was a polymorphism in the fourth intron of PDCD1,^[24]^ the substitution of T for C in the enhancer within the intron might disrupt the binding site of RUNX1, alter the regulation of gene expression, and influence the PD-1 pathway.^[24]^ PDCD-1.3 (rs11568821) polymorphism may impair the inhibitory effect of PD-1 and thus may lead to positive regulation of cytotoxic lymphocyte activity in T allele carriers.^[5,24]^ Thus, variant TC genotype might contribute to decrease risk of cancer. However, the exact mechanisms are still needed to be analyzed in future studies. However, 11 Asian studies were included in our meta-analysis, and the majority were studies performed in China. Race might play an important role in deriving the conclusions of the current meta-analysis. Some studies have a bigger sample size compared with others within 1 analysis, which might also generate bias. This suggests that the results should be explained with caution.

The problem of heterogeneity and publication bias, which may influence the results of meta-analyses, should also be explained. Significant heterogeneity existed in the analysis among 3 polymorphisms. The heterogeneity might result from cancer types, ethnicity, and the source of controls. However, due to the limited number of studies included, we did not perform analysis of these factors based on subgroups. Publication bias is another important issue in meta-analyses. In the present study, publication bias was analyzed by using Begg funnel plots and the Egger test. We did not detect a significant publication bias for all polymorphisms, suggesting the reliability of our results.

This meta-analysis has pooled the available data from the eligible studies, which has significantly increased the statistical power. However, there are still some weaknesses. First, cancer is a multifactorial disease from complex interactions between environmental exposure and genetic factors. In this meta-analysis, we had insufficient data to conduct an evaluation of such interactions for the role of *PDCD1* polymorphisms and factors in cancer development. Second, numerous present studies are limited for some polymorphisms only. Thus, investigations involving large number of different ethnicities are necessary for a more reliable assessment on their associations. Third, the heterogeneity between studies exists in some polymorphisms, and that may affect the stability of the results.

In conclusion, our meta-analysis suggests that PDCD-1.5 (rs2227981) and PDCD-1.3 (rs11568821) polymorphisms are associated with susceptibility to cancer, while rs2227982, rs7421861, and rs10204525 polymorphism may not be associated with cancer risk. These results should be interpreted cautiously. In order to better understand the potential roles of *PDCD1* polymorphisms in cancer, further studies with larger sample sizes, combining genetic and other environmental risk factors, are needed.

## Supplementary Material

Supplemental Digital Content
